# Cognitive, biomarker, and neuroimaging indices associated with traumatic encephalopathy syndrome across two independent athlete cohorts

**DOI:** 10.21203/rs.3.rs-9385305/v1

**Published:** 2026-04-28

**Authors:** Brooke Conway Kleven, Lung-Chang Chien, Dana Surwill, Michael L. Alosco, Jennifer V. Wethe, Yorghos Tripodis, Charles H. Adler, Martha E. Shenton, Ofer Pasternak, Douglas I. Katz, Elaine Peskind, Laura J. Balcer, Inga K. Koerte, Jesse Mez, Eric M. Reiman, Robert C. Cantu, Robert A. Stern, Henrik Zetterberg, Charles Bernick, Jeffrey L. Cummings

**Affiliations:** University of Nevada, Las Vegas; University of Nevada, Las Vegas; University of Nevada, Las Vegas; Boston University; Mayo Clinic; Boston University; Mayo Clinic; Harvard University; Harvard University; Boston University; University of Washington; New York University; Ludwig-Maximilians-Universität München; Boston University; Banner Health; Boston University; Boston University; University of Gothenburg; Lou Ruvo Brain Institute; University of Nevada, Las Vegas

**Keywords:** Chronic traumatic encephalopathy, Traumatic encephalopathy syndrome, repetitive head impacts, blood biomarkers, neuroimaging, neurodegeneration

## Abstract

**Background:**

Traumatic encephalopathy syndrome (TES) is a clinical research construct used to identify individuals at risk for chronic traumatic encephalopathy (CTE) following exposure to repetitive head impacts (RHI). Adjudication of TES relies on clinical features such as progressive cognitive impairment and neurobehavioral dysregulation. Blood-based biomarkers and structural neuroimaging abnormalities have been associated with TES but are not part of the criteria. This study evaluated whether TES identification was associated with the combined contribution of cognitive performance, blood biomarkers, and structural neuroimaging measures across two well-characterized cohorts.

**Methods:**

Participants included 158 professional fighters from the Professional Athletes Brain Health Study and 149 former American football players from The DIAGNOSE CTE Research Project. Three indices were constructed representing complementary domains: a cognitive index reflecting cohort-specific cognitive features, a blood biomarker index including plasma neurofilament light chain, glial fibrillary acidic protein, total tau, tau phosphorylated at amino acid 231, and *APOE*-ε4 carrier status, and an imaging index comprising volumetric MRI measures of subcortical structures, ventricles, and corpus callosum subregions. Grouped weighted quantile sum regression models were estimated within each cohort to evaluate associations between these indices and TES while adjusting for age, race, competition status, and RHI exposure.

**Results:**

Multidomain models demonstrated improved model performance compared with single-domain models in both cohorts (PABHS: AUC=0.91, PPV=0.80; DIAGNOSE CTE: AUC=0.84, PPV=0.85). Biomarker and imaging indices contributed additional information across cohorts, although imaging contributions were more prominent in fighters whereas blood biomarker associations were stronger in football players.

**Conclusion:**

TES in RHI-exposed athletes was associated with a convergent clinicobiological profile observed across two independent cohorts with distinct exposure patterns. These findings support multidomain analytic frameworks for evaluating correlated biological signals in RHI-exposed populations and may inform future studies of TES and CTE.

## Introduction

The long-term effects of repetitive head impacts (RHI) raise concerns regarding contact sports, including the risk of developing chronic traumatic encephalopathy (CTE)^1^, a neurodegenerative disorder associated with RHI exposure. While the standard for CTE diagnosis is a post-mortem neuropathologic confirmation of hyperphosphorylated tau (pTau) deposits at the depths of cortical sulci and around small blood vessels, the National Institute of Neurological Disorders and Stroke (NINDS) developed consensus diagnostic criteria for traumatic encephalopathy syndrome (TES) to assist in research involving living individuals with a history of RHI and potential CTE.^2^ The criteria for TES adjudication follows a stepwise process requiring an individual to have substantial exposure to RHI, core clinical features of neurobehavioral dysregulation and/or cognitive impairment, a progressive course of symptoms, and no other medical condition(s) that can fully account for the symptoms.^2^

Research evaluating the validity of TES has explored contributions of various biomarkers that may support the earlier diagnosis of CTE or serve as a method to track the progression of neurodegenerative processes that may occur following RHI exposure. Blood biomarkers such as neurofilament light chain protein (NfL), which serves as a marker of axonal injury, and glial fibrillary acidic protein (GFAP), indicative of activated astroglia, have been associated with a diagnosis of TES.^3–5^ Magnetic resonance imaging (MRI) indicates that TES is frequently associated with subcortical and medial temporal lobe atrophy, with volumetric loss observed in regions such as the thalamus, hippocampus, and corpus callosum.^6–8^

Biomarker studies typically evaluate markers individually rather than integrating multiple biological domains into a composite framework.^3,6,15–18^ The purpose of the current study was to determine whether TES identification is characterized by the combined contribution of cognitive performance, blood biomarkers, and structural neuroimaging measures across two independent cohorts. Because cognitive impairment is currently a core feature of the TES diagnostic criteria, the cognitive domain in the present study represents the clinical component of the syndrome. We therefore hypothesized that individuals identified as TES+ would demonstrate a convergent pattern of cognitive dysfunction, biological abnormalities, and neuroanatomical changes, and that biological domains would contribute to TES identification beyond the clinical domain alone. To evaluate this hypothesis, we applied an integrative modeling framework to quantify how individual and combined domains align with TES identification.^19^ We leveraged data from two independent athlete cohorts, the Professional Athletes Brain Health Study (PABHS; consisting primarily of boxers and mixed martial arts fighters)^20^ and the Diagnostics, Imaging, and Genetics Network for the Objective Study and Evaluation of Chronic Traumatic Encephalopathy (DIAGNOSE CTE; consisting of former professional and college football players)^21^ to determine whether multimodal associations with TES are reproducible across athlete populations with distinct patterns of RHI exposure and demographic characteristics.

## Methods

Details of the PABHS cohort have been previously published.^20^ The PABHS is a longitudinal cohort of over 900 professional athletes with a history of RHI exposure and age/sex-matched unexposed controls. To meet the cohort’s criteria of a retired fighter, athletes must not have plans for future fights and cannot have competed in a sanctioned fight within the past two years. Active fighters must have competed in a professionally sanctioned event within the past two years and must wait at least 45 days following a fight to attend baseline and/or annual visits. For any participant with multiple visits, only the data from the most recent visit were included in the current analyses. RHI exposure was measured using the number of professional fights completed. Consistent with prior analyses from PABHS, a minimum threshold of 10 professional fights was used to operationalize substantial exposure to RHI within this cohort. This threshold was selected to represent sustained professional-level combat exposure and to exclude individuals with limited professional experience whose fight histories may not reflect consistent RHI exposure. In combat sports, the number of professional fights provides a more direct proxy for cumulative head impacts than years of participation alone, as bout frequency, rounds per fight, and intensity of exposure vary substantially across athletes.

Methods for the DIAGNOSE CTE have been detailed previously.^22^ Briefly, this eight-year longitudinal study enrolled 240 men ages 45–74, including 120 former professional football players (PRO), 60 former college football players (COL), and 60 unexposed asymptomatic men. The asymptomatic men were not included in this study. The PRO participants were required to have played 12 or more years of organized football, including at least three years in college and more than three years in the National Football League (NFL). COL participants were required to have played six or more years of American football, with at least three years at the college level.^13,23^

## TES Adjudication

TES status was determined independently for each cohort through multidisciplinary diagnostic consensus conferences using the NINDS Consensus Diagnostic Criteria for TES.^2^ To be identified as TES+ required confirmation of substantial RHI exposure, evaluation of core clinical features including cognitive impairment and/or neurobehavioral dysregulation, evidence of progressive symptom worsening, and determination that symptoms were not fully explained by other neurological or psychiatric conditions. “Substantial exposure” reflects a history of RHI from activities such as contact sports or military service, typically involving prolonged participation or roles associated with frequent head impacts.^2^ All participants included in the present analyses (TES + and TES-) met cohort-specific predefined thresholds for substantial exposure as outlined above. As such, RHI exposure did not distinguish TES+ from TES- within the analytic sample and was not used to define case status in the present analyses.

Cognitive impairment was uniformly defined based on reported decline from prior functioning and performance at least 1.5 standard deviations below normative expectations on formal neuropsychological testing, with impairment required in episodic memory or executive function. As the neuropsychological batteries differed between cohorts and included different numbers and types of tests, cognitive impairment was operationalized using comparable criteria across studies. In DIAGNOSE CTE, participants were assigned provisional levels of certainty for CTE pathology (suggestive, possible, or probable) based on TES criteria and supportive clinical features. The same TES adjudication framework was applied in PABHS; however, provisional levels of certainty for CTE pathology were not assigned in that cohort.

## Study Population

Participants from both cohorts with critical missing data were excluded. To maintain comparability between cohorts, participants with TES based solely on neurobehavioral dysregulation without evidence of cognitive impairment were excluded from the present analyses. Although neurobehavioral dysregulation was considered during TES adjudication in both cohorts, the methods used to ascertain these symptoms differed between studies, including the use of certain instruments, informant, or study partner reports in DIAGNOSE CTE that were not available in PABHS. The resulting analytic sample therefore included TES-positive (TES+) participants with cognitive features and TES-negative (TES-) participants across both cohorts. Participants in both cohorts provided written informed consent prior to any study procedures at their respective study sites.

## Study Variables

To evaluate whether TES is characterized by convergence across clinical and biological domains, we constructed three complementary predictor indices representing cognitive function, blood-based biomarkers, and volumetric MRI regions:

### Cognitive Index.

Cognitive impairment is central to TES and required for possible or probable CTE diagnoses. The cognitive index was constructed to reflect TES-relevant cognitive features while accommodating cohort-specific neuropsychological batteries. Given that cognitive impairment contributes to TES adjudication, some measures included in the cognitive index overlap with those used to define TES classification; accordingly, the cognitive index represents the clinical domain of the syndrome rather than an independent predictor. Because the specific instruments differed across cohorts, cognitive variables were harmonized at the domain level (e.g., memory, processing speed, executive function) rather than by individual tests. Standardized performance metrics were used to represent each domain, and indices were derived and analyzed separately within each cohort. Comparisons between cohorts were therefore performed at the level of overall model patterns rather than pooled variable-level data.

For PABHS, cognitive performance was derived from standardized computer-based assessments administered through CNS Vital Signs and the Cleveland Clinic Concussion App (C3 Logix), supplemented by a semantic verbal fluency task using Animal Naming.^24,25^ The PABHS cognitive index encompassed seven variables: educational attainment as a marker of cognitive reserve, verbal memory, verbal fluency, processing speed, psychomotor speed, reaction time, and executive control expressed through speeded task performance.

For DIAGNOSE CTE, cognitive performance was derived from a comprehensive neuropsychological battery spanning attention, psychomotor speed, executive function, learning and memory, language, and visuospatial ability.^26^ Raw scores were converted to age, sex, and/or education-adjusted T-scores using established normative data.^13^ For the present analyses, the DIAGNOSE CTE cognitive index incorporated six variables: educational attainment, verbal memory, verbal fluency, attention, executive function, and psychomotor or processing speed.

Processing speed, psychomotor speed, and reaction time represent related but distinct components of cognitive function and motor-cognitive integration. Impairments in these activities have been reported in RHI-exposed cohorts, including in samples characterized using TES criteria.^6,14^ These domains were included to capture broader cognitive variability for analytic comparison and were not used for TES adjudication. Specifically, because cognitive impairment is a core feature of TES, the cognitive index is not independent of the outcome. Therefore, sensitivity analyses excluding the cognitive index were conducted to evaluate whether biomarker and imaging domains independently associated with TES.

### Blood Biomarker Index.

Plasma biomarkers selected as predictors in this analysis included NfL, GFAP, total tau protein, and tau protein phosphorylated at threonine 231 (pTau231). These biomarkers were selected a priori based on prior literature implicating neuroaxonal injury, glial response, and tau-related processes in RHI and neurodegenerative risk. To ensure analytic consistency, only biomarkers measured in both PABHS and DIAGNOSE CTE were included in the present index. Apolipoprotein E (*APOE*) genotype was determined for all PABHS and DIAGNOSE CTE participants at baseline to identify the presence of the *APOE*-ε*4* allele.

Blood biomarkers were measured using Single molecule array (Simoa) technology on an HD-X platform (Quanterix, Billerica, MA, USA) at the Clinical Neurochemistry Laboratory, Sahlgrenska University Hospital, Mölndal, Sweden by laboratory technicians who were blinded to the clinical data. These samples, while analyzed on the same platform, were performed in separate batches among the cohorts. Batch-bridging using internal quality control samples ascertained comparability of the measurements. A full description of the sample collection process was previously published for both cohorts.^21,27,28^ The blood biomarker index comprised a mixture of five predictors, including plasma total tau, pTau231, GFAP, NfL, and a dichotomous *APOE*-ε*4* carrier status.

### Imaging Index.

High-resolution T1-weighted anatomical MRIs were performed at a single timepoint for DIAGNOSE CTE participants and at each annual visit for PABHS athletes. In accordance with the methodology described above, for participants with multiple visits, only the scan from the most recent visit was included in the present analyses to ensure temporal alignment across study variables. For PABHS, all imaging was acquired at a single site. DIAGNOSE CTE imaging was acquired across multiple sites using harmonized acquisition protocols.^22^ The Siemens Skyra with a 32-channel head coil was used to acquire structural 3D T1-weighted magnetization-prepared rapid acquisition gradient echo images across both cohorts. Imaging data underwent quality control using FreeSurfer quality analysis tools (FreeSurfer 5.3 QATools, 2021) and visual inspection of cortical and subcortical segmentations. Reconstructions that did not meet predefined quality thresholds, including a minimum signal-to-noise ratio of 16, were reprocessed according to standardized procedures. No scans from either cohort were excluded in the current analysis based on quality control criteria.

Fifteen regions of interest were pre-specified for the imaging index based on prior epidemiological and pathological studies.^1,14,17,29,30^ These included averaged left and right subcortical gray matter volumes (thalamus, caudate, hippocampus, putamen, amygdala, pallidum), ventricular volumes (lateral ventricles, inferior lateral ventricles, third ventricle, fourth ventricle), and five anatomically distinct corpus callosum subregions (posterior, mid-posterior, central, mid-anterior, anterior). The corpus callosum was subdivided to capture regional vulnerability of interhemispheric white matter tracts to RHI. Volumetric regions were calculated using FreeSurfer’s automated full-brain segmentation process (version v.6; FreeSurfer, Boston, MA). Mean values of hemispherical volumes were calculated to create an average of left and right cerebral and ventricular regions. To ensure consistent directionality of volumetric predictors, ventricular volumes were multiplied by −1, as increased ventricular size reflects greater atrophy and should therefore correspond to lower structural integrity consistent with reductions in subcortical volumes. Imaging features were analyzed within cohort-specific models rather than pooled across cohorts.

## Data Analysis

Initial evaluations included descriptive statistics of the study populations and comparisons between TES groups using t-tests for continuous variables and Fisher-Freeman-Halton test for categorical variables. Because predictors were measured on different scales, all variables were converted to percentile ranks prior to analysis to place predictors on a common scale and reduce the influence of extreme values during mixture component estimation. For imaging variables, volumetric measures were adjusted for intracranial volume prior to percentile transformation to account for individual differences in head size.

We performed uni-index and multi-index generalized weighted quantile sum (GWQS) regressions to examine how the cognitive, blood biomarker, and imaging predictors were associated with TES individually and simultaneously within each cohort. Indices were constructed by transforming individual variables within each domain into quantiles and combining them into a single weighted index, with weights estimated through bootstrap sampling to reflect the relative contribution of each variable to the overall association with TES. The adjusted multi-index models included age, race, competition status (active vs retired; collegiate vs professional), and RHI exposure proxy (number of professional fights or years of football participation). Sex was included as a covariate in PABHS only, as all DIAGNOSE CTE participants were male. This is presented in the equation below:

$$\text{Logit}(\text{Y}=1)=\beta_{0}+\theta_{\text{2}}{\text{WQS}}_{2}+\theta_{3}{\text{WQS}}_{3}+{\text{Y}}_{1}{\text{Z}}_{1}+{\text{Y}}_{2}{\text{Z}}_{2}+{\text{Y}}_{3}{\text{Z}}_{3}+{\text{Y}}_{4}{\text{Y}}_{4}+{\text{Y}}_{5}{\text{Y}}_{5}$$

where Y is the binary outcome of TES (negative = 0; positive = 1), (WQS_1_, WQS_2_, WQS_3_) are the grouped cognitive, blood biomarker, and imaging indices, (θ_1_, θ_2_, θ_3_) are the regression coefficients representing the effect of each index, β_0_ is the model intercept, and (γ_1_, γ_2_, γ_3_, γ_4_, γ_5_) are the regression slopes for the covariates (age, sex, race, RHI exposure, status). Note that the model for DIAGNOSE CTE does not include γ_5_ Z_5_.

Each model used 1000 bootstrap iterations to improve the stability of the estimated mixture weights, with final weights defined as the average across bootstrap samples. The estimated coefficients of each index were obtained using logistic regression with maximum likelihood estimation. Because both multi-index models suffered from quasi-complete separation, the Firth correction was utilized to resolve this modeling issue.^31^ Odds ratios (ORs) were calculated as the exponentiated regression coefficients. The contributions of individual variables within each index were assessed through empirically estimated weights. A larger weight indicated a greater contribution from each variable to the corresponding index. These weights reflect the relative contribution of correlated predictors to model discrimination within the GWQS framework and should not be interpreted as evidence of biological hierarchy, causal influence, or mechanistic pathways. To aid interpretation, a threshold value (τ), defined as the reciprocal of the number of variables within each index, was used to identify variables contributing more than would be expected under equal weighting (cognitive index τ ≈ 0.17; blood biomarker index τ = 0.20; imaging index τ ≈ 0.07). To assess whether associations between biological measures and TES were independent of the cognitive index, sensitivity analyses were conducted using models including only biomarker and imaging indices. Model performance was evaluated using discrimination and classification metrics, including the area under the receiver operating characteristic curve (AUC), sensitivity, specificity, and positive predictive value (PPV). Calibration was additionally assessed using smoothed calibration plots and the Brier score. Analyses were conducted in RStudio 2025.05.0 (RStudio, PBC, Massachusetts), with statistical significance defined as p < 0.05.

## Results

This analysis consisted of 307 participants, including 158 professional fighters (35% racial minority, 14% female) from the PABHS and 149 American football players from DIAGNOSE CTE (33% racial minority, 0% female). Among all participants, 51% (n=158) were identified as TES+ (PABHS=41%; DIAGNOSE CTE=63%). Across cohorts, TES+ participants had greater RHI exposure, lower educational attainment, and were more likely to be retired (PABHS) or former professional-level (DIAGNOSE CTE) athletes compared to TES- (**Table 1**). All DIAGNOSE CTE participants were ≥45 years of age (p-value=0.7010), whereas 61% of participants in PABHS were younger than 45 years (p-value=0.0010). There were no significant differences between biological sex within the TES groups for the PABHS athletes (p-value=0.8470). Sex differences were not evaluated in DIAGNOSE CTE, as all participants were male. A full description of cohort characteristics and index-level classifications are provided in **Table 1**.

In PABHS, the adjusted multi-index model including age, race, sex, competition status (active vs retired), and RHI exposure (number of professional fights) as covariates demonstrated excellent discrimination of TES (AUC=0.91), whereas discrimination remained strong but more modest in DIAGNOSE CTE (AUC=0.84). At the Youden-optimal threshold, the PABHS model achieved a sensitivity of 0.91 and specificity of 0.84 (PPV=0.80). In DIAGNOSE CTE, sensitivity was 0.84 and specificity was 0.73 (PPV=0.85). Calibration analyses demonstrated good agreement between predicted probabilities and observed TES status in both cohorts, with Brier scores of 0.11 in PABHS and 0.15 in DIAGNOSE CTE (**Additional file 1: Figure A1**).

### PABHS

In the PABHS cohort, each index was significantly associated with TES in uni-index models (Cognitive AUC=0.85/PPV=0.71; Biomarker AUC=0.62/PPV=0.69; Imaging AUC=0.81/PPV=0.78). The AUC and PPV values for the unadjusted multi-index model, however, performed better than the uni-index models. In the unadjusted multi-index model, higher values (improved scores) of the cognitive index were associated with 75% decreased odds of TES (OR=0.25; 95% confidence interval [CI]=0.16, 0.37; p-value<.0001) and higher values of the imaging index were associated with 70% lower odds of TES (OR=0.30; 95% CI=0.20, 0.43; p-value<.0001). The blood biomarker index did not significantly influence TES status in the unadjusted model.

The adjusted multi-index model indicated the best fit for this dataset (AUC=0.91/PPV=0.80), as it demonstrated improved discrimination compared with individual indices. In the adjusted model, higher values of the cognitive index remained strongly associated with lower odds of TES (OR=0.25; 95% CI=0.14, 0.42; p-value<.0001) while the imaging index independently contributed additional discriminatory information, with a 65% lower odds of TES per unit increase (OR=0.35; 95% CI=0.17, 0.65; p-value=0.0015). Female sex (OR=9.36; 95% CI=2.44, 40.10; p-value=0.0014), older age (OR=4.01; 95% CI=1.41, 11.94; p-value=0.0101), and greater RHI exposure (OR=1.05; 95% CI=1.02, 1.08; p-value=0.0016) all significantly predicted the odds of TES (**Table 2**). This estimate should be interpreted cautiously given the small percentage of female participants. Similar to the unadjusted model, the blood biomarker index did not significantly influence TES status in the adjusted model.

**Figure 1:**
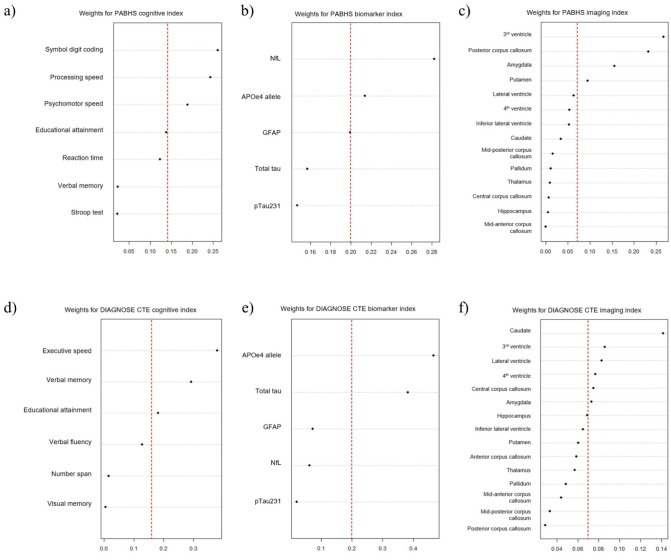
Estimated weights of predictors in the a) PABHS cognitive index, b) PABHS blood biomarker index, c) PABHS imaging index, d) DIAGNOSE CTE cognitive index, e) DIAGNOSE CTE blood biomarker index, and f) DIAGNOSE CTE imaging index among the adjusted multi-index models for both cohorts. The red lines indicate the τ values, where τ = 0.14 and 0.17 for the cognitive index (PABHS and DIAGNOSE CTE, respectively), 0.20 for the blood biomarker index, and 0.07 for the imaging index.

The estimated weights for individual predictor variables in the adjusted multi-index model are shown in Figure 1. In the cognitive index, the following predictors had a weight greater than τ=0.14: symbol digit coding (0.27), processing speed (0.26), and psychomotor speed (0.24). In the blood biomarker index, the following predictors had a weight greater than τ=0.20: NfL (0.29), *APOE*-ε4 allele (0.22), and GFAP (0.20). In the imaging index, the following predictors had a weight greater than τ=0.07: volumes of 3^rd^ ventricle (0.29), posterior corpus callosum (0.24), amygdala (0.16), and putamen (0.09).

### DIAGNOSE CTE

In the DIAGNOSE CTE cohort, each index demonstrated significant association with TES in uni-index models (Cognitive AUC=0.78/PPV=0.84; Biomarker AUC=0.62/PPV=0.76; Imaging AUC=0.62/PPV=0.74). The AUC and PPV values were improved in the unadjusted multi-index model. In the unadjusted multi-index model, higher values of the cognitive index were associated with 83% lower odds of TES (OR=0.17; 95% CI=0.08, 0.31; p-value<.0001) while higher values of the blood biomarker index were associated with a 107% increase in the odds of TES (OR=2.07; 95% CI=1.32, 3.38; p-value=0.0023). The imaging index did not significantly influence TES status in the unadjusted model.

The adjusted multi-index model including age, race, competition status (college vs professional), and RHI exposure (years of football) as covariates indicated the best fit for this dataset (AUC=0.84; PPV=0.84). Similar to PABHS findings, analysis of the data supported our hypothesis that TES is best characterized by a multidomain profile, as integration of cognitive, biomarker, and imaging indices improved model discrimination beyond any single domain alone. In the adjusted model, the cognitive index remained strongly associated with lower odds of TES (OR=0.15; 95% CI=0.07, 0.30; p-value<.0001) while the blood biomarker index independently contributed additional discriminatory information, with a 125% increase in the odds of TES per unit increase (OR=2.25; 95% CI=1.39, 3.83; p-value=0.0015) (**Table 2**). RHI exposure (OR=1.22; 95% CI=1.06, 1.43; p-value=0.0081) significantly predicted the odds of TES, while competition status and age did not. Similar to the unadjusted model, the imaging index did not significantly influence TES status in the adjusted model.

In the cognitive index, the following predictors had a weight greater than τ=0.17: executive speed (0.37), verbal memory (0.29), and educational attainment (0.19). In the blood biomarker index, the following predictors had a weight greater than τ=0.20: *APOE*-ε4 allele (0.41) and total tau (0.38). In the imaging index, the following predictors had a weight greater than τ=0.07: caudate (0.17), 3^rd^ ventricle (0.09), lateral ventricle (0.08), 4^th^ ventricle (0.07), central corpus callosum (0.07), and amygdala (0.07). Summary statistics and visualization of the weighted cognitive, blood biomarker, and imaging indices by TES status are provided in Appendix (**Figure A2** and **Table A3)**, demonstrating distinct separation in index distributions between TES+ and TES- participants across both cohorts.

### Sensitivity Analysis: Biomarker and Imaging Indices Without Cognitive Index

To evaluate whether associations between biological measures and TES were independent of the cognitive index, a sensitivity analysis, visualized in Table 3, was conducted including only biomarker and imaging indices.

In PABHS, the unadjusted model demonstrated a significant association for the imaging index (OR=0.28; 95% CI=0.18, 0.42; p-value<.0001), whereas the blood biomarker index was not associated with TES status. After adjustment, both indices were significantly associated with TES (biomarker index OR=2.06; 95% CI=1.06, 4.28; p-value=0.0455; imaging index OR=0.18, 95% CI=0.09, 0.35; p-value<.0001), with strong model discrimination (AUC=0.88; PPV=0.72).

In DIAGNOSE CTE, both indices were associated with TES in the unadjusted model (biomarker index OR=1.60; 95% CI=1.08, 2.42, p-value=0.0204; imaging index OR=0.60; 95% CI=0.37, 0.94; p-value=0.0277). These associations remained significant in adjusted models (biomarker index OR=1.65; 95% CI=1.01, 2.74; p-value=0.0486; imaging index OR=0.48; 95% CI=0.26, 0.84; p-value=0.0133), with modest model discrimination (AUC=0.71; PPV=0.78). Overall, results were consistent with the primary analyses. Although the adjusted multidomain model incorporating cognitive, biomarker, and imaging indices demonstrated the strongest discrimination of TES, biomarker and imaging indices remained significantly associated with TES when the cognitive index was excluded from the model.

## Discussion

By integrating cognitive, imaging, and blood biomarker data from two independent cohorts of athletes exposed to RHI, this study demonstrates consistent associations between TES and combined cognitive, biomarker, and neuroimaging indices across two independent cohorts. Across both cohorts, multi-index models demonstrated improved model performance compared with single-domain models, with adjusted AUC values of 0.91 in PABHS and 0.84 in DIAGNOSE CTE. Even when the cognitive index was excluded, blood biomarker and imaging indices remained associated with TES across both cohorts, indicating that objective biological measures contribute substantial information beyond clinical features alone.

Although the overall pattern of findings was consistent across cohorts, the relative contribution of biological domains differed. Structural imaging features contributed more prominently to model discrimination among professional fighters (PABHS), whereas blood biomarker signals were more strongly associated with TES among former football players (DIAGNOSE CTE). These differences may reflect variation in cumulative injury burden, age distribution, or – in cases where CTE is present – stage of neurodegenerative progression between cohorts. Together, these findings support the concept that TES reflects a broader clinicobiological phenotype rather than an exclusively symptom-defined construct. Likewise, the reproducibility of the outcomes in two groups of athletes with unique RHI exposures increases the credibility and applicability of the results. The GWQS framework complements other multivariate approaches such as the Global Statistical Test (GST) by empirically weighting correlated predictors to quantify their relative contributions to the overall association. While GST provides a robust global measure of group-level differences, GWQS adds variable-level interpretability, allowing clearer identification of the factors most strongly linked to diagnostic status.^19^ This approach is promising for risk stratification, cohort enrichment, and experimental therapeutic efforts in populations exposed to RHI.

Our results demonstrate the value of utilizing an integrated approach over individual measures for identifying individuals with TES who might eventually be shown to have CTE. We hypothesize that athletes with TES that incorporates fluid biomarker and imaging changes are more likely to have CTE than those without these concomitant indicators of brain pathology.

Our findings reinforce previous literature suggesting that cognitive deficits associated with RHI exposure are diverse while also identifying cognitive features common to both cohorts.^6,17,32,33^ In both cohorts’ cognitive indices, processing speed exceeded the τ threshold, indicating a prominent role in differentiating TES + and TES- status among both groups. These results are consistent with prior studies that have identified a decline in processing speed as associated with neurodegeneration related to RHI exposure.^17^ Verbal memory was more heavily weighted in the retired football players than the fighters. This may reflect the more precise testing and quantification of neurocognitive assessments in the DIAGNOSE CTE cohort. The inverse association between educational attainment and TES supports cognitive reserve theory, in which higher education serves as a protective factor against clinical symptom expression despite potential underlying pathology.^34^

While GWQS-derived weights do not imply causality or biological mechanism, the relative prominence of specific biomarkers across cohorts provides a descriptive framework for contextualizing existing biological literature. The biomarker indices revealed *APOE*-ε4 allele received the highest relative weight within the blood biomarker index in both cohorts, along with NfL and GFAP in fighters, whereas former football players showed elevated total tau. These findings are consistent with prior literature identifying these markers as associated with astroglial activation, axonal damage, and neurodegeneration in RHI-exposed populations.^1,35–37^ Many of the PABHS participants are under the age of 45, which may contribute to lower tau burden than the DIAGNOSE CTE participants. Furthermore, the observed differences between cohorts may reflect not only methodological and demographic factors, but also the distinct biomechanical forces experienced in combat sports versus football. Impacts in combat sports often involve rotational acceleration and focal blows, potentially producing different injury patterns or affecting distinct brain regions compared to the linear and repetitive collisions common in football. While variability in imaging indices may partly result from differences in scanner platforms and cohort age, the potential influence of sport-specific exposure mechanics warrants further investigation.

The consistent prominence of the *APOE*-ε*4* carrier across both cohorts underscores its potential role in the identification of TES risk. Present in approximately 60–70% of individuals with AD, *APOE*-ε*4* is well established as a genetic risk factor that promotes amyloid deposition and accelerates tau-related neurodegeneration.^38^ *APOE*-ε*4* has also been linked to heightened inflammatory responses, including increased activation of astrocytes and microglia through impaired triglyceride metabolism.^39^ These mechanistic pathways align with our broader findings, in which *APOE*-ε*4* clustered with elevated GFAP may be indicative of a neuroinflammatory response. Recent neuropathological evidence has demonstrated that *APOE*-ε*4* is associated with greater severity of CTE pathology, supporting a role for this allele in modifying disease progression following RHI exposure.^40^ In the context of RHI, *APOE*-ε*4* may amplify vulnerability to both proteinopathies and inflammation, leading to degeneration and subsequent clinical decline.

The current diagnostic criteria for TES depend solely on clinical domains.^2^ These criteria rely on symptom reporting and neuropsychological performance, which may reflect heterogeneous processes, may not capture early biological changes, and may overlap with other neurological or psychiatric conditions. In addition, the clinical data required for TES adjudication often involve comprehensive neuropsychological evaluation and longitudinal clinical assessment, which can be resource-intensive and may limit scalability. Integrating biologic data with clinical observations represents one approach to resolving this dilemma. In contrast, the biological measures included in the multi-index model, such as blood biomarkers and structural MRI, can be obtained using standardized protocols and may offer more scalable and objective approaches to monitoring brain health. Specifically, the multi-index model developed here can serve as a stratification tool for identifying high-risk individuals or as an inclusion criterion for clinical trials investigating disease targeted therapies. The improved positive predictive values (PPV = 80 and 85%, respectively) underscore the potential to reduce heterogeneity and improve cohort stratification for such trials.

This study had several strengths, including a large sample size (N = 307) and reproducibility of findings from two cohorts of professional athletes with substantial RHI exposure. Several limitations are acknowledged. Although the TES criteria applied here are currently the best available, the lack of pathological confirmation restricts our ability to validate and directly link index performance to underlying CTE pathology. The statistical modeling in this study, while valuable for identifying individual and cumulative weights, limits inferences about causality or the progression of the syndrome. The DIAGNOSE CTE cohort was restricted to individuals aged 45 years and older, whereas the PABHS cohort included many younger participants. Age differences influence both biomarker expression and neurodegenerative burden. Furthermore, some of the cognitive measures applied normative values derived from general population samples that differ from the demographic and educational profiles of the PABHS cohort. As the PABHS participants generally have lower educational attainment than the DIAGNOSE CTE participants, applying standard normative corrections may have led to misidentification or overestimation of impairment. The absence of behavioral information in the PABHS cohort may have influenced the diagnosis and characteristics of TES identified in this cohort. The p-tau measure available was p-tau231 and other p-tau species may be more informative. Our findings should be interpreted as descriptive and hypothesis-generating with respect to TES identification. Future studies should evaluate the independent contribution of neurobehavioral dysregulation to TES in cohorts where these symptoms are systematically measured. Such analyses may help clarify whether neurobehavioral features contribute meaningfully to multidomain TES profiles or represent a distinct clinical dimension of RHI exposure.

## Conclusion

TES in RHI-exposed athletes was associated with a convergent clinicobiological profile across two independent cohorts. In both cohorts, multidomain models integrating cognitive, biomarker, and neuroimaging indices demonstrated stronger discrimination of TES than single-domain models. Biomarker and imaging indices contributed additional information across cohorts, although imaging contributions were more prominent in fighters while biomarker associations were stronger in football players. These findings support multidomain analytic frameworks for examining biological signals associated with TES and may inform future studies aimed at improving risk stratification for CTE.

## Supplementary Material

Tables 1 to 3 are available in the Supplementary Files section.

Supplementary Files

This is a list of supplementary files associated with this preprint. Click to download.

- Appendix.docx
- Tables.docx

## Data Availability

The datasets used and/or analyzed during the current study are available from the corresponding author upon reasonable request.
